# Supplementary material for: Evaluating the theoretical performance of aircraft wastewater monitoring as a tool for SARS-CoV-2 surveillance
Source: PLOS Glob Public Health. 2023 Jun 22;3(6):e0001975. doi: 10.1371/journal.pgph.0001975 (PMC10286959; doi:10.1371/journal.pgph.0001975)
Supplement: Attachment — Submitted filename: reviewer_response_letter.docx [file pgph.0001975.s001.docx]

Dear Editor,

Thankyou for considering our article titled *Evaluating the theoretical performance of aircraft wastewater monitoring as a tool for SARS-CoV-2 surveillance.* We would also like to thank the reviewers for their useful comments and suggestions, which have been addressed in this letter and, where appropriate, in the revised article.

The original comments and questions from reviewers are in *red italics* in this letter, with our response given directly below each point in *black italics* and any reference to changes made to the manuscript in ***bold italics***. All line numbers provided refer to line numbers in the clean (untracked) revised manuscript. Simple spelling/grammar corrections have not been directly addressed in this letter but corrections have been made in the main manuscript.

**Reviewer #1:** 
*ABSTRACT:
-In the final section of the ABSTRACT, I would suggest adding a sentence or two that states that in the Discussion section of the paper the authors provide insights on the suitability of aircraft wastewater monitoring based on a number of parameters, including sample availability, sample collection, test sensitivity and the characteristics of the target pathogen.(Lines 269 to 320.) This would allow the reader to assess the described computer modeling exercise in the broader context of infection disease surveillance.*

***Lines 31-34 now add more detail on the insights provided by the modelling. We have mentioned the relationship between detection of a pathogen in wastewater and the defecation probability, faecal shedding probability and limit of detection.***

*INTRODUCTION:
In the opening section, the authors focus on the role of aircraft travel in disease dissemination. It may be useful to include a sentence or two on other cross border settings, such as in mainland Europe, where a preponderance of cross border travel may not be by air (for example, train travel)
Line 70, I would suggest deleting the word "case" in the sentence,"....One such use case......."
Line 93, please replace the word, "effect" with "effective."*

**Lines 97-107 now discuss other cross-border transmission routes. We have referenced a study looking at detection in cruise ship wastewater and discussed possible dissemination via land routes.**

**Reviewer #2**
*- You compare the analysis of wastewater with the swabs taken on arrival. I did not understand in your text the status of the passengers who would be tested by swab: random selection? Symptomatic passengers? Passengers who would report contact with a case? Because the probability of being infected, and therefore the probability of being positive, varies significantly in these three groups. In practice, many territories carried out either a systematic screening before departure, or a screening after arrival of symptomatic persons. Random screening is not, to my knowledge, a common practice.*

***We have now clarified that we are comparing wastewater monitoring to random sample swabbing of passengers (lines 111-120). We have added several references demonstrating the use of random sample swabbing (or voluntary swabbing) at borders.*** *In the UK, India, and Japan voluntary and/or random sampling was used to test passengers arriving from China in late 2022 and early 2023 as a response to the lifting of restrictions in China and the risk of exposure to new SARS-CoV-2 variants. Voluntary swabbing has been used in 4 airports in the US during 2022 and 2023. We have assumed that voluntary swabbing and random sample swabbing are equivalent (i.e., the probability of volunteering for a swab is independent of a person’s infection status).*

*We have not considered systematic pre-departure screening of patients as this fulfils a different purpose to aircraft wastewater monitoring. Pre-departure screening and post-arrival symptomatic screening both aim to prevent the onward transmission of a disease (or variant of a disease) in the destination country. The use of wastewater proposed in this paper is as a monitoring tool, rather than a containment and control tool. As such it would be unhelpful to compare wastewater monitoring with systematic pre-departure screening.* ***We have now clarified this point in lines 75-89 in which we have discussed the uses and limitations of aircraft wastewater monitoring.***

*We anticipate wastewater monitoring to have the highest value when other forms of screening are not available. Widespread pre-departure screening is now uncommon, and so we do not feel it would be helpful to assess the value of wastewater monitoring within a context of ongoing pre-departure screening.*

*- You mention the fact that wastewater testing is cost effective compared to screening on arrival (under certain conditions of proportion of screened passengers and sensitivity of the test). I did not find any mention of cost analysis of the two measures in your methods section. Although intuitive, this statement does not seem to be supported by your results. Even if the costs of the analyses may vary from country to country, I think you could introduce in your work estimates made on the basis of the cost in UK, complementing in a simple way your results. (Or you could could remove the mention about cost-effectiveness)*

***Lines 89-96 now discuss the cost of aircraft wastewater monitoring in more detail.*** *We have not provided a full cost analysis of the two types of testing as this is commercially sensitive information. We have included a reference to a paper which compares the cost of municipal wastewater monitoring to clinical testing.*

*In general, it seems to me that the most important issue is really not discussed in your article: what would be the objective, the benefit of such a screening? In the case of pre-departure screening, the objective was to prevent the travel of infectious subjects, reducing the probability of transmission during the flight and in the territory of arrival; in the case of arrival screening (systematic or of symptomatic or at-risk individuals) the objective was the quarantine of positive travellers (and contact tracing of on-board neighbouring persons), with the objective of reducing transmission in the country of arrival. These methods have significant costs (in financial and human terms), and important limitations (in terms of effective case detection capacity), but meet clear prevention objectives. What would be the benefit of wastewater testing? I would argue that it is not possible to carry out targeted prevention measures on the basis of these results. So it would be a way to estimate the risk of introduction for "purely" epidemiological purposes? (In this case, Sars-CoV2 might be the wrong example, because it is no more an emerging pathogen.) What kind of actions could be taken after a positive result?
I strongly suggest that you expand the introduction and discussion by making more explicit the (public health) rationale for the use of aircraft wastewater monitoring, and the context in which you think it would be relevant to use it.*

***Lines 75-89 now provide a more detailed description of the public health rationale for aircraft wastewater monitoring****. In short, we consider two uses:*

1. *Estimation of the point-in-time of the incursion of a pathogen (or variant of a pathogen) into a country. This would provide valuable insight to disease modellers and epidemiologists to assess the potential impact of a disease.*
2. *International surveillance and risk assessment. Monitoring of aircraft wastewater would help to build a clearer picture of global disease prevalence. This is particularly useful for low-and-middle income countries where national surveillance may be limited.*

***We have also clarified that wastewater monitoring is not a suitable tool to aid in the containment and contact tracing of positive results (lines 75-78)****. As such we do not anticipate there being a particular response to a positive result, beyond the notification of public health authorities.*

*We disagree with the suggestion that SARS-CoV-2 is an inappropriate pathogen for this type of analysis. While it is no longer and emerging pathogen, new variants of the disease still pose significant risk. Aircraft wastewater monitoring may help public health authorities to estimate when a recently identified variant entered a country, and could help us to understand the global spread of a new variant in countries with limited national surveillance.*

**Reviewer #3**

*The grammar in the entire manuscript has to be improved greatly. In the methodology section, there is a need to include a description of the study, that is, the two countries used for the simulation and predictions. There is indiscriminate use of italics and future tenses in the method section.*

***We have revised the manuscript to address any potential grammar mistakes****. We hope that the reviewer is satisfied with the style of the revised version. Italics have been used in the original submission for section headings, mathematical symbols and figure captions.*

*We use the UK as a destination country for simulation and analysis, although this is largely irrelevant as we assume an entirely susceptible population and only consider the early part of the epidemic. The choice of country only effects the population size – a factor which will have almost no influence while case numbers are low. For similar reasons we do not specify a seed country, and instead use a generic population of 10^6^ people.* ***This has been clarified in the manuscript (line 152).*** *The choice of country may affect the passenger numbers and flights times. Hence, we consider a range for these parameters in the first part of the analysis. The number of daily flights would also vary with country choice, however as this is equivalent to the number of passengers in the model we do not consider it as a separate variable.*

We hope that the clarifications and amendments described above constitute a satisfactory response to the reviewers. We look forward to hearing from you.

Yours sincerely,

Joseph Shingleton (corresponding author)
